# Occult Pulmonary Micrometastases From a Papillary Thyroid Carcinoma Undetectable by Imaging

**DOI:** 10.7759/cureus.103328

**Published:** 2026-02-09

**Authors:** Risa Hashimoto, Yoshiro Otsuki, Toru Nakamura

**Affiliations:** 1 General Thoracic Surgery, Seirei Hamamatsu General Hospital, Hamamatsu, JPN; 2 Pathology, Seirei Hamamatsu General Hospital, Hamamatsu, JPN

**Keywords:** micrometastasis, papillary thyroid carcinoma, pulmonary metastasis, radioactive iodine scanning, video-assisted thoracoscopic surgery

## Abstract

Pulmonary metastases from papillary thyroid carcinoma (PTC) are usually detectable by computed tomography (CT) or radioactive iodine (RAI) scanning. Pathologically proven pulmonary micrometastases that are completely undetectable by imaging have rarely been reported. A 39-year-old woman underwent surveillance CT four years after total thyroidectomy for PTC. A solitary right pulmonary nodule, which had been present since before thyroid surgery and had gradually enlarged, was clinically suspected to be metastatic. Wedge resection of the lung revealed the nodule to be a benign hemangioma. However, careful histopathological examination incidentally identified multiple tiny nodules measuring 200-1,000 μm in the surrounding lung parenchyma. These lesions exhibited characteristic features of PTC and were positive for thyroglobulin on immunohistochemistry, confirming pulmonary micrometastases. A retrospective CT review and postoperative RAI scintigraphy failed to detect these lesions. The patient received RAI therapy and remains disease-free 126 months after pulmonary resection. This case demonstrates that pulmonary micrometastases from PTC may exist despite the absence of radiological or scintigraphic findings. Occult micrometastatic disease may be more prevalent than clinically appreciated, highlighting a diagnostic limitation of imaging-based surveillance.

## Introduction

Papillary thyroid carcinoma (PTC) generally has a favorable prognosis, and clinically latent lesions are frequently identified incidentally as microcarcinomas during surgery for other head and neck malignancies or at autopsy [1,2]. Despite its indolent nature, distant metastases do occur, with the lung being one of the most common sites [3,4]. Pulmonary metastases from PTC are typically identified using computed tomography (CT) or radioactive iodine (RAI) scintigraphy. However, pathologically confirmed pulmonary micrometastases that are completely undetectable by these imaging modalities have not been well documented. We report a rare case in which pulmonary micrometastases from PTC were incidentally discovered during surgical resection of a radiologically visible but benign lung nodule.

## Case presentation

**Figure 1:**
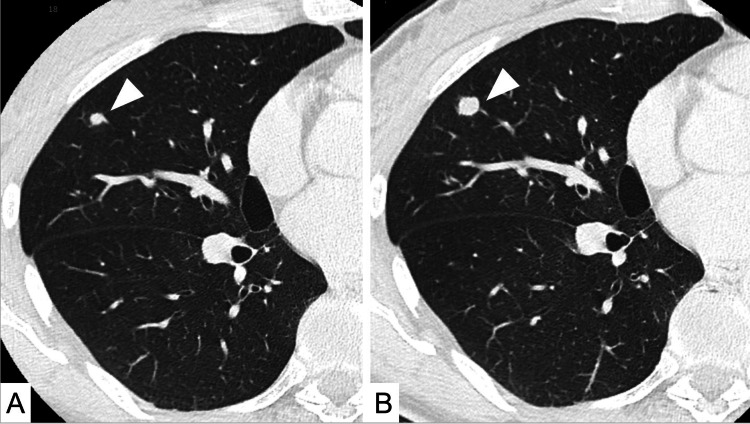
Chest CT findings. (A) Plain axial CT image showing a 5.8 mm nodule in the right middle lobe (arrowhead). (B) The nodule enlarged to 9 mm over four years (arrowhead).

A 39-year-old woman was referred for evaluation of an enlarging pulmonary nodule detected on surveillance CT. She had undergone total thyroidectomy followed by postoperative RAI therapy for PTC four years earlier. Her serum thyroglobulin level before treatment was 1.5 ng/mL (reference range: <33.70 ng/mL) and remained within the normal range (0.5-2.2 ng/mL) during the follow-up period. At that time, a solitary right pulmonary nodule had already been identified and was clinically suspected to represent pulmonary metastasis, although pathological confirmation had not been obtained. Over a four-year period, the nodule gradually increased in size from 5.8 mm to 9 mm without evidence of additional metastatic disease (Figure 1).

**Figure 2:**
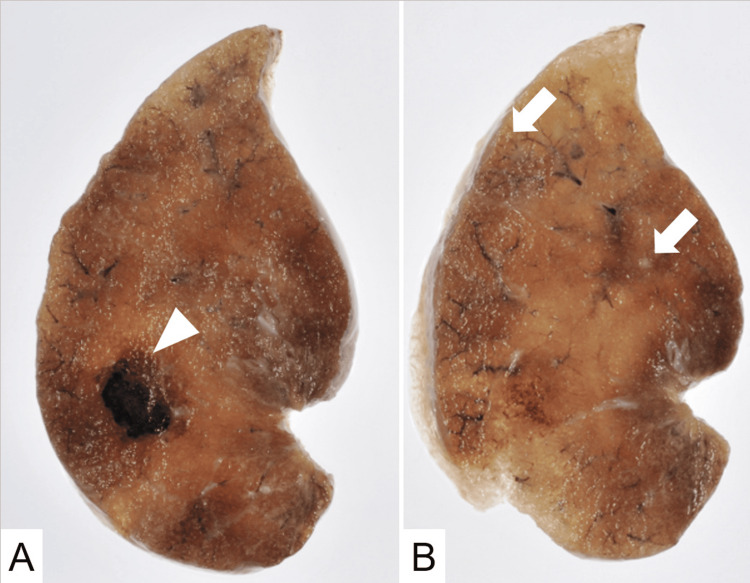
Gross findings of the surgical specimen. (A) A well-circumscribed nodule is shown. (B) Tiny nodules (arrows) were found in the surrounding lung parenchyma.

**Figure 3:**
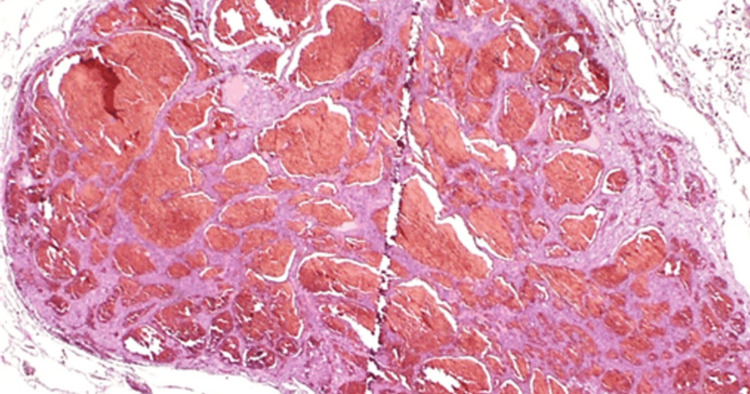
Histopathological finding of the target lesion. The pathological finding of the target lesion revealed a hemangioma without any malignant findings (hematoxylin and eosin staining, ×20).

Given the interval growth and solitary nature, a wedge resection of the right middle lobe was performed for curative intent, despite a low preoperative serum thyroglobulin level of 0.5 ng/mL. The postoperative course was uneventful. Gross examination of the resected specimen revealed a well-circumscribed pulmonary nodule (Figure 2A). Histopathological analysis demonstrated that the lesion was a hemangioma with no malignant features (Figure 3). Notably, careful examination of the surrounding lung parenchyma revealed several tiny nodules measuring 200-1,000 μm in diameter (Figure 2B).

**Figure 4:**
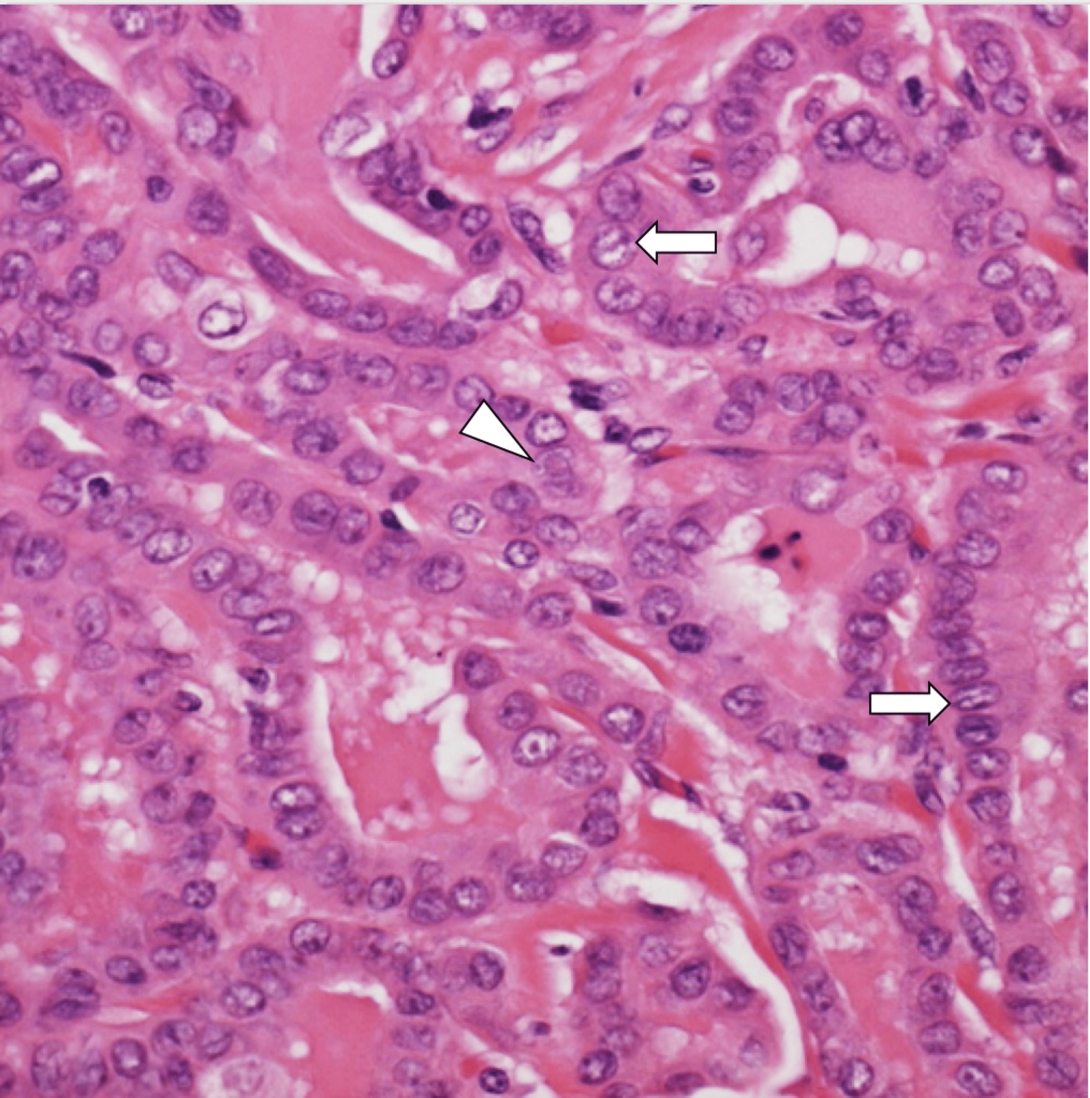
Histopathological finding of the tiny nodule. The pathological finding of the satellite nodules revealed a papillary growth pattern with nuclear longitudinal grooves (white arrow) and eosinophilic intranuclear inclusions (arrowhead) (hematoxylin and eosin staining, ×400).

**Figure 5:**
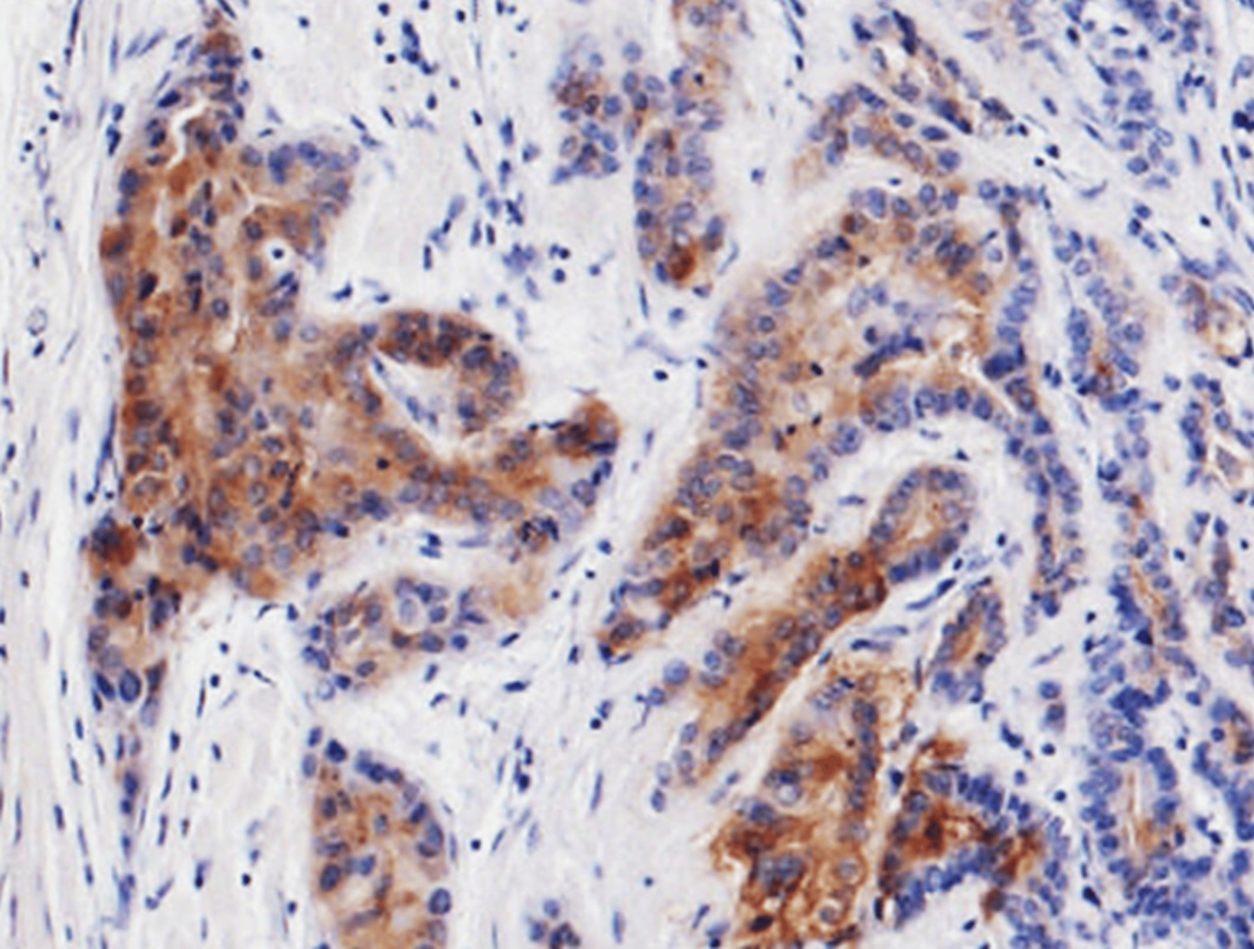
Immunohistochemical staining of the tiny nodule. Immunostaining for thyroglobulin was positive for tumor cells (peroxidase staining, ×100).

These nodules exhibited papillary architecture with atypical cuboidal cells, nuclear grooves, and eosinophilic intranuclear inclusions, findings characteristic of PTC (Figure 4). Immunohistochemical staining for thyroglobulin was positive, confirming their origin as metastatic PTC (Figure 5).

A retrospective review of preoperative CT images failed to identify these micrometastatic lesions. Additionally, postoperative RAI scintigraphy showed no abnormal uptake suggestive of metastatic disease. The patient subsequently received radioactive iodine-131 therapy (3.7 GBq). She remains clinically and radiologically disease-free 126 months after pulmonary resection, with a serum thyroglobulin level of 4.98 ng/mL within the normal range. Anti-thyroglobulin antibodies were negative throughout the follow-up period.

## Discussion

This case highlights a rare but clinically important phenomenon: pulmonary micrometastases from PTC that are completely undetectable by conventional imaging modalities. While pulmonary metastases from PTC are well recognized, they are generally identified by CT or RAI scintigraphy. In contrast, the micrometastatic lesions in this case were discovered only through meticulous pathological examination of surgically resected lung tissue. Thyroid microcarcinomas, defined as tumors measuring ≤10 mm, are frequently detected incidentally and are usually associated with an excellent prognosis. Nevertheless, distant metastases have been reported even in clinically indolent cases, although their true prevalence remains uncertain [5,6]. The present case suggests that occult pulmonary micrometastases may be underrecognized due to inherent limitations in radiological resolution. Early identification of distant metastases and timely administration of RAI therapy have been associated with improved outcomes in differentiated thyroid carcinoma [5,7]. In our patient, micrometastases were detected at a subclinical stage, and subsequent RAI therapy may have contributed to favorable disease control. However, the prognostic significance of such occult micrometastases remains unclear. The prevalence of papillary thyroid microcarcinomas detected by ultrasonography has been reported to be markedly higher than that of clinically apparent disease [8]. Analogously, micrometastatic pulmonary involvement may be more common than previously appreciated, remaining clinically silent and radiologically invisible. This case underscores a diagnostic blind spot in imaging-based surveillance strategies for thyroid carcinoma.

## Conclusions

We report a rare case of occult pulmonary micrometastases from PTC incidentally identified during surgical resection of a benign lung lesion. Pulmonary micrometastases may exist despite the absence of radiological or scintigraphic findings. Awareness of this possibility may help refine postoperative surveillance and management strategies for patients with PTC.
